# Effect of Metformin Treatment on Lipoprotein Subfractions in Non-Diabetic Patients with Acute Myocardial Infarction: A Glycometabolic Intervention as Adjunct to Primary Coronary Intervention in ST Elevation Myocardial Infarction (GIPS-III) Trial

**DOI:** 10.1371/journal.pone.0145719

**Published:** 2016-01-25

**Authors:** Ruben N. Eppinga, Minke H. T. Hartman, Dirk J. van Veldhuisen, Chris P. H. Lexis, Margery A. Connelly, Erik Lipsic, Iwan C. C. van der Horst, Pim van der Harst, Robin P. F. Dullaart

**Affiliations:** 1 University of Groningen, University Medical Center Groningen, the Department of Cardiology, Groningen, the Netherlands; 2 LabCorp, Raleigh, North Carolina, United States of America; 3 University of Groningen, University Medical Center Groningen, the Department of Critical Care, Groningen, the Netherlands; 4 University of Groningen, University Medical Center Groningen, the Department of Endocrinology, Groningen, the Netherlands; Sapienza University of Rome, ITALY

## Abstract

**Objective:**

Metformin affects low density lipoprotein (LDL) and high density (HDL) subfractions in the context of impaired glucose tolerance, but its effects in the setting of acute myocardial infarction (MI) are unknown. We determined whether metformin administration affects lipoprotein subfractions 4 months after ST-segment elevation MI (STEMI). Second, we assessed associations of lipoprotein subfractions with left ventricular ejection fraction (LVEF) and infarct size 4 months after STEMI.

**Methods:**

371 participants without known diabetes participating in the GIPS-III trial, a placebo controlled, double-blind randomized trial studying the effect of metformin (500 mg bid) during 4 months after primary percutaneous coronary intervention for STEMI were included of whom 317 completed follow-up (clinicaltrial.gov Identifier: NCT01217307). Lipoprotein subfractions were measured using nuclear magnetic resonance spectroscopy at presentation, 24 hours and 4 months after STEMI. (Apo)lipoprotein measures were obtained during acute STEMI and 4 months post-STEMI. LVEF and infarct size were measured by cardiac magnetic resonance imaging.

**Results:**

Metformin treatment slightly decreased LDL cholesterol levels (adjusted *P* = 0.01), whereas apoB remained unchanged. Large LDL particles and LDL size were also decreased after metformin treatment (adjusted *P*<0.001). After adjustment for covariates, increased small HDL particles at 24 hours after STEMI predicted higher LVEF (*P* = 0.005). In addition, increased medium-sized VLDL particles at the same time point predicted a smaller infarct size (*P*<0.001).

**Conclusion:**

LDL cholesterol and large LDL particles were decreased during 4 months treatment with metformin started early after MI. Higher small HDL and medium VLDL particle concentrations are associated with favorable LVEF and infarct size.

## Introduction

The clinical relevance of plasma lipids and lipoprotein levels in predicting (recurrent) coronary heart disease is well appreciated [1]. Indeed, pharmacological treatment aimed at lowering low density lipoprotein (LDL) cholesterol is an essential part of the routine care of patients with a history of myocardial infarction (MI) [2]. Importantly, lipoprotein particles are highly heterogeneous in size, structure and function with probable consequences for cardiovascular risk prediction [3–10]. In the non-acute setting, LDL and high density lipoprotein (HDL) particle characteristics have been proposed to be more closely associated with (incident) coronary heart disease compared to LDL cholesterol and HDL cholesterol concentrations [3–5,7–9,11–21]. When determined at presentation of MI, LDL cholesterol, HDL cholesterol and triglycerides have been variably shown to predict recurrent adverse cardiac events [10]. However, little is currently known about the prognostic value of lipoprotein subfraction characteristics obtained in the setting of an acute MI.

In subjects with impaired glucose tolerance, metformin administration modestly reduces the LDL particle concentration, and concomitantly decreases small dense LDL particles and increases small HDL particles, as determined by nuclear magnetic resonance (NMR) spectrometry [22]. Furthermore, metformin improves insulin resistance [23,24], which has been recently identified as a marker of adverse cardiac outcome [24,25]. Taken together these findings provide a rationale to determine whether metformin affects lipoprotein subfraction characteristics in patients with an acute coronary event.

The Glycometabolic Intervention in Adjunct to Primary Percutaneous Coronary Intervention in ST-Segment Elevation Myocardial Infarction (GIPS-III) trial was designed to evaluate the effect of 4 months metformin treatment on left ventricular function in non-diabetic patients with ST-segment elevation MI (STEMI) [26,27]. The rationale of this study is based on experimental findings showing that metformin may beneficially affect left ventricular function through activation of a number of intracellular pathways and alters mitochondrial function as outlined extensively elsewhere [26]. Among other potentially beneficial effects, metformin may also affect lipid and lipoprotein levels [26], which was a predetermined tertiary efficacy endpoint of the GIPS-III trial [26]. This randomized trial provides a framework to determine effects of metformin on lipoprotein metabolism, and to evaluate associations of lipoprotein subfractions, obtained in the setting of an acute MI, with left ventricular ejection fraction (LVEF) and infarct size assessed at 4 months.

The present anciliary analyses were initiated to test the extent to which metformin treatment affects lipoprotein subfraction characteristics in GIPS-III participants. Second, we determined the association of lipoprotein subfractions with LVEF and infarct size.

## Methods

### Study population

The GIPS-III trial is has been registered as clinical trial with identifier: NCT01217307. The design and primary results of the GIPS-III trial has been reported in detail elsewhere [26,27]. The inclusion and exclusion criteria of the GIPS-III stuy are reported in Table 1. In brief, 380 non-diabetic patients undergoing primary percutaneous coronary intervention (PCI) for STEMI were randomized to receive a 4-month regimen with either metformin 500 mg twice daily or matching placebo twice daily. During the PCI procedure, all patients except one provided verbal informed consent followed by written informed consent. This subject was excluded, as well as were subjects in whom lipoprotein subfractions measurements were not available. As a result, 185 subjects receiving metformin and in 186 subjects receiving placebo were available for the current analyses. From these participants we determined lipoprotein subfractions in 371 subjects at baseline, 338 subjects 24 hours post-MI and 317 subjects 4 months post-MI. A total of 271 subjects completed 4 months follow-up evaluation by cardiac magnetic resonance imaging (MRI). From these subjects in 268 lipoprotein subfractions were determined at baseline, 250 subjects 24 hours post-MI and 257 4 subjects months post-MI.

**Table 1:** In- and exclusion criteria for the GIPS-III trial.

Inclusion criteria	Exclusion criteria
The diagnosis acute MI defined by chest pain suggestive for myocardial ischemia for at least 30 min, the time from onset of the symptoms less than 12 h before hospital admission, and an ECG recording with ST- segment elevation of more than 0.1 mV in 2 or more leads	Prior MI
Verbal followed by written informed consent	Diabetes
At least one stent sized ≥ 3.0 mm	Creatinin >177 μ mol/L measured pre-PCI
Eligible for cardiac MRI-scan:	Need for coronary artery bypass grafting
Body Mass Index <40 kg/m2 an estimated life-expectancy of less than 6 months	Rescue PCI after thrombolytic therapy
no ferromagnetic metal objects in the body	When subjects develop a condition which, in the investigator’s judgment, precludes study therapy
no claustrophobia	Inability to provide informed consent
	Younger than 18 years
	Contra-indication to metformin
	an estimated life-expectancy of less than 6 months

Abberviations: MI: myocardial infarction; ECG: electrocardiogram; PCI: percutaneous coronary intervention; MRI: magnetic resonance imaging

Blood samples were obtained shortly after admission at the catheterization laboratory, after 24 hours post-MI and 4 months after randomization. Very low density lipoproteins (VLDL), LDL and HDL particle profiles were determined at these 3 time points. Samples for glucose, glycated hemoglobin (HbA1c), plasma total cholesterol, LDL cholesterol, HDL cholesterol, triglycerides, apolipoprotein (apo)B and apoA-I were obtained at the catheterization laboratory and 4 months after randomization.

### Laboratory Measurements

Serum and EDTA-anticoagulated plasma samples were stored at − 80°C until analyzed. Plasma total cholesterol, LDL cholesterol and HDL cholesterol were measured by a direct quantitative assay using cholesterol (PEG-) esterase and (PEG-) cholesterol oxidase on a Roche Modular *P* autoanalyzer (Roche Diagnostics, Indianapolis, IN, USA). Non-HDL cholesterol was calculated as the difference between total cholesterol and HDL cholesterol. Triglycerides (TG) were quantified using the LipoProfile-3 algorithm (LipoProfile-3 algorithm; LipoScience Inc. (now Labcorp Inc.), Raleigh, North Carolina, USA) [16]. Quantification of TG was accomplished by converting NMR particle numbers to lipid mass concentration units, assuming that the lipoprotein particles have normal lipid content. NMR-derived values correlate well with chemically measured values. Apolipoprotein (apo) B and apoA-I were computationally estimated by the use of the high-throughput 1H nuclear magnetic resonance (NMR) metabolomics platform of Computational Medicine (Oulu, Finland) [28].

VLDL, LDL and HDL particle profiles were measured by NMR spectroscopy with the LipoProfile-3 algorithm (LabCorp, Raleigh, North Carolina, USA), as described [16]. VLDL, LDL and HDL subclasses were quantified from the amplitudes of their spectroscopically distinct lipid methyl group NMR signals, and were expressed in concentration units, i.e. μmol/L or nmol/L. The lipoprotein subfraction particle concentrations are considered to represent an estimate of the respective lipoprotein particle numbers. Diameter range estimates were for VLDL: large VLDL (including chylomicrons if present; > 60 nm), medium VLDL (35 to 60 nm) and small VLDL (27 to 35 nm), for LDL: IDL (23 to 27 nm), large LDL (21.2 to 23 nm) and small LDL (18 to 21.2 nm), and for HDL: large HDL particles: 9.4 to 14 nm; medium HDL particles: 8.2 to 9.4 nm; small HDL particles: 7.3–8.2 nm. The VLDL, LDL and HDL particle concentrations were calculated as the sum of the respective lipoprotein subclasses. Weighted-average VLDL, LDL and HDL sizes were derived from the sum of the diameter of each subclass multiplied by its relative mass percentage based on the amplitude of its methyl NMR signal [16].

NT-proBNP was routinely measured with a sandwich immunoassay on a Roche Modular E platform (Mannheim, Germany).

### Cardiac Magnetic Resonance Imaging (MRI)

LVEF and infarct size were measured by cardiac magnetic imaging [29]. These outcome measures were assessed by MRI four months after infarction. Details of the imaging analysis has been reported elsewhere [26,27]. An independent core laboratory (Image Analysis Center, Free University Medical Center, Amsterdam, The Netherlands) evaluated the MRI scans and assessed the primary efficacy measure, blinded for treatment allocation and clinical patient data.

### Myocardial blush grade (MBG)

MBG was categorized as previously described [30]. A physician blinded to data analyzed coronary angiograms.

### Statistical analysis

R (version 3.02 or higher, http://www.r-project.org/) was used for statistical analyses. Values for continuous variables that are normally distributed are presented as mean ± SD. Continuous variables not normally distributed are presented as median and interquartile ranges (IQRs).

Because not all lipoprotein subfractions were normally distributed (Shapiro-Wilk Normality test, *P* < 0.05), they were normalized using rank-based inverse normal transformation across all time points.

Pearson correlation coefficients were calculated from lipoprotein subfractions at 4 months after acute MI, and plotted using the corrplot function of the corrplot package of R. The correlation matrix is presented in S1 Fig.

The extent to which clinical parameters, laboratory values and lipoprotein subfraction measurements between treatment (metformin and placebo) groups were significantly different at the various time-points (baseline, i.e. at admission for MI, 24 hours post-MI and 4 months post-MI) was determined after data normalization using unpaired T tests. Difference in medication use was assessed using a multinomial chi-squared test. None of the baseline lipoprotein variables were significantly different between the treatment groups.

For this reason the statistical comparisons of in the main results were given as the *P*-values of the unpaired T tests after 4 months of treatment in primary analysis. In addition, regression models were used to examine the changes in lipoprotein subfractions between the two treatment groups (placebo and metformin). In this analysis, the respective lipoprotein variable at 24 hours and after 4 months was the dependent variable with the following independent covariates: treatment assignment, age at randomization, sex, body mass index (BMI), statin use at 4 months and the baseline lipoprotein subfraction of interest.

For routine laboratory values the significance level was set at *P* ≤ 0.01. In view of multiple testing of lipoprotein subfraction data, a principal components (PCs) analysis was carried out using the prcomp-function of R. The first 8 components explained 96% of the variation, of which the first 5 components explained 84% of the variation in the data set (S1 File). On the basis of the PCs, the multiple testing corrected significance level of lipoprotein subfractions was set to *P* ≤ 0.05/8 components, equivalent to *P* ≤ 0.0063.

To examine the relationship between baseline laboratory values or lipoprotein subfraction levels at different time points (baseline and 24 hours post-MI) with LVEF or infarct size (4 months post-MI), linear regression models were used. In the primary analysis, LVEF or infarct size was the dependent variable with baseline laboratory values or lipoprotein subfractions as the independent variable. A secondary analysis was performed adjusting for baseline laboratory values or lipoprotein subfractions, age at randomization, sex, baseline NT-proBNP concentration, treatment allocation, MBG and statin use at 4 months which we considered to be relevant covariates.

## Results

### Clinical paramaters, MRI parameters and laboratory values

Table 2 summarizes clinical parameters, MRI parameters and laboratory measurements at baseline and at 4 months after intervention. There were no significant differences in clinical and laboratory characteristics between the two treatment groups at baseline. After 4 months of intervention there was a significantly lower LDL cholesterol in the metformin group (2.1 [1.8–2.4] mmol/L) group compared to the placebo group (2.2 [1.8–2.4 2.7] mmol/L); *P* = 0.01 after adjustment for baseline LDL cholesterol, age at randomization, sex, BMI, and statin use at 4 months). In a sensitivity analysis we performed a logistic regression with reduction of LDL cholesterol as dependent variable and metformin as independent variable; this also resulted in a trend towards lower LDL cholesterol (β = -0.25; SE: 0.12; *P* = 0.04).

**Table 2:** Clinical-, MRI- and laboratory parameters by treatment group at baseline and after 4 months.

	Baseline (Myocardial Infarction)				4 Months Treatment			
**Clinical parameters**	**Placebo (*n* = 186)**	**Metformin (*n* = 185)**	***P***		**Placebo (*n* = 160)**	**Metformin (*n* = 157)**	***P***	P**
Age, mean	58.72 ± 11.44	58.80 ± 11.82	0.95					
Women, n (%)	48 (26%)	46 (25%)	0.83					
Body Mass index, mean	27.0 ± 3.9	27.0 ± 3.8	0.92		**27.4 ± 4.0**	**27.0 ± 4.0**	0.37	
Statin use, n (%)	15 (8%)	13 (7%)	0.70					
**Medication use**	**at discharge**		0.25		**at 4 months**		0.92	
Statin, n (%)	185 (99%)	184 (99%)			150 (94%)	147 (94%)		
Aspirin, n (%)	181 (97%)	178 (96%)			154 (96%)	146 (93%)		
Thienopyridine, n (%)	186 (100%)	185 (100%)			157 (98%)	152 (97%)		
Coumarine, n (%)	7 (4%)	13 (7%)			7 (4%)	12 (8%)		
Beta-blocker, n (%)	181 (97%)	174 (94%)			150 (94%)	148 (94%)		
ACE-inhibitor or ARB, n (%)	141 (76%)	153 (83%)			139 (87%)	140 (89%)		
Calcium-channel blocker, n (%)	3 (2%)	9 (5%)			13 (8%)	11 (7%)		
Aldosterone antagonist, n (%)	12 (7%)	25 (14%)			19 (12%)	25 (16%)		
**Laboratory values**			***P***	P*			***P***	P**
Glucose (mmol/L)	8.4 (7.0–9.8)	8.2 (7.0–9.4)	0.93	0.93	5.6 (5.2–6.1)	5.6 (5.1–6.2)	0.94	0.90
HbA1c (%)	5.8 (5.6–6.0)	5.8 (5.6–6.1)	0.51	0.43	5.9 (5.7–6.1)	5.9 (5.6–6.0)	0.11	0.07
Creatinine (μmol/L)	72 (62.3–80)	72 (61.0–84.0)	0.66	0.69	79.5 (72–88.3)	79 (70.0–87.0)	0.65	0.49
Total Cholesterol (mmol/L)	5.4 (4.8–6.0)	5.3 (4.7–6.1)	0.87	0.87	3.9 (3.5–4.4)	3.8 (3.4–4.2)	0.07	0.06
LDL Cholesterol (mmol/L)	3.8 (3.3–4.4)	3.7 (3.1–4.4)	0.57	0.65	**2.2 (1.8–2.7)**	**2.1 (1.8–2.4)**	**0.02**	**0.01**
non-HDL Cholesterol (mmol/L)	4.2 (3.7–4.9)	4.2 (3.6–4.9)	0.98	0.97	2.7 (2.7–3.2)	2.6 (2.2–3.0)	0.16	0.11
HDL Cholesterol (mmol/L)	1.1 (0.9–1.3)	1.1 (0.9–1.3)	0.94	0.97	1.2 (1.0–1.5)	1.1 (0.9–1.4)	0.14	0.09
Triglycerides (mmol/L)	0.69 (0.57–0.88)	0.69 (0.59–0.98)	0.22	0.20	1.38 (1.04–1.84)	1.44 (1.02–2.01)	0.12	0.31
ApoB (g/L)	0.79 (0.68–0.93)	0.80 (0.64–0.93)	0.87	0.95	0.76 (0.66–0.86)	0.74 (0.66–0.87)	0.68	0.48
ApoA-I (g/L)	1.26 (1.16–1.40)	1.29 (1.16–1.42)	0.38	0.36	1.33 (1.23–1.47)	1.30 (1.21–1.44)	0.11	0.17
NT-proBNP (ng/L)	78 (37–175)	84 (42–235)	0.31	0.27	167 (74–355)	163 (67–389)	0.47	0.65
**Cardiac MRI parameters**								
LVEF, % (95% CI)	NA	NA			54.7 (53.4–56.1)	53 (51.5–54.6)	0.10	
LVEDV, ml (95% CI)	NA	NA			194.1 (186.4–201.9)	194.6 (186.5–202.6)	0.94	
LVESV, ml (95% CI)	NA	NA			89.8 (83.8–95.8)	93.6 (87.3–100.0)	0.39	
LVEDM, g (95% CI)	NA	NA			101.6 (97.2–106.0)	102.0 (98.1–105.8)	0.91	
Infarct size, % (95% CI)	NA	NA			8.8 (7.5–10.1)	8.8 (7.5–10.2)	0.97	

Abbreviations: Apo: apolipoprotein; ARB: aldosterone receptor blocker; BMI: body mass index; HbA1c: glycosylated hemoglobin; HDL: high density lipoproteins; LDL: low density lipoproteins; LVEF: left ventricular ejection fraction; LVEDV: left venticular end diastolic volume; LVESV: left ventricular end systolic volume; LVEDM: left ventricular end diastolic mass; non-HDL: non-high density lipoproteins; NT-proBNP: N-terminal pro brain natriuretic peptide. Baseline and 4 months data are presented as mean ± SD, median (interquartile range), and number (percentage) as appropriate. *P*-values for placebo vs. Metformin by unpaired T tests. *P*-value* adjusted for age at randomization, sex, BMI. *P*-value** additionally adjusted for respective baseline laboratory value and statin use at 4 months. Bold is *P*-value ≤ 0.01.

Plasma triglycerides were much lower in both groups at baseline compared to the values after 4 months of follow-up (*P* < 0.001 for all comparisons), whereas total cholesterol, non-HDL cholesterol, LDL cholesterol and apoB were higher at baseline than after follow-up of 4 months (*P* < 0.001 for all comparisons).

### Effect of metformin treatment on lipoprotein subfractions

**Fig 1:**
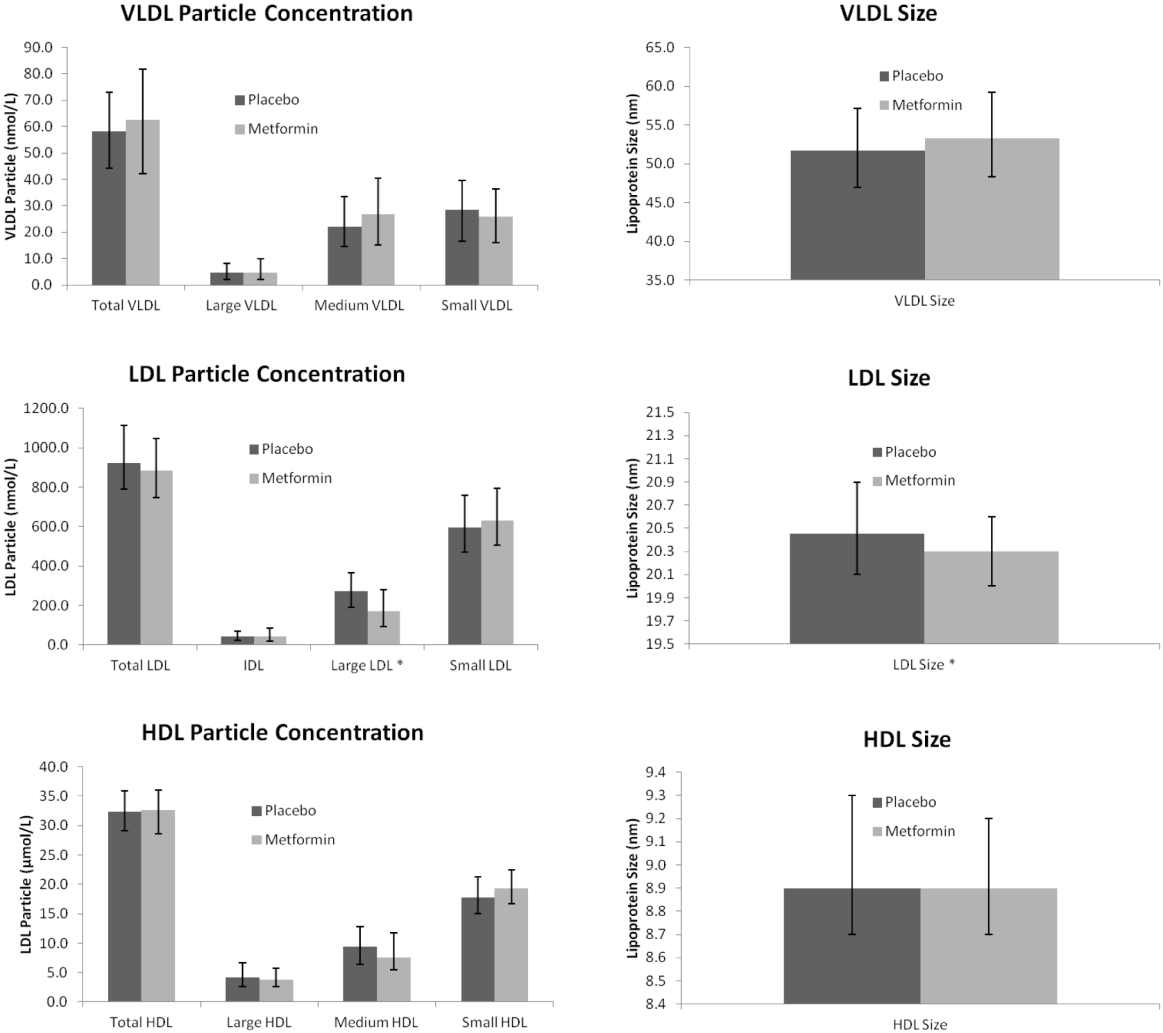
Lipoprotein subfraction concentrations and size using NMR after 4 months according to treatment group (VLDL-*P*; LDL-*P*; HDL-*P*; Lipoprotein size). Data are presented as median (interquartile range). *P*-values from unpaired—tests. *P*-value ≤ 0.0063 placebo vs. Metformin group.

Table 3 and S2 Fig show the median values of lipoprotein subfraction levels and lipoprotein sizes at baseline, 24 hours post-MI and 4 months post-MI. There were no significant differences between the two treatment groups at baseline and 24 hours post-MI. After 4 months of treatment, large LDL particles (270.5 [190.0–365.8] vs 170.0 [93.0–278] nmol/L and LDL size (20.3 [20.0–20.6] vs 20.5 [20.1–20.9] nm) were decreased in the metformin group compared to the placebo group (*P* ≤ 0.001 for each). After correcting for their baseline values, age at randomization, sex, BMI and statin use at 4 months, these differences remained significant *P* ≤ 0.001 for each). Fig 1 illustrates the changes in the various lipoprotein subfractions on lipoprotein particle size after 4 months of therapy with metformin compared to placebo. Remarkably, all VLDL subfractions and VLDL size were much lower in both groups at baseline compared to 24 hours and after 4 months of follow-up (*P* < 0.001 for all comparisons). Conversely, the LDL particle concentration, as well as the IDL and large LDL subfractions were higher at baseline than at after 4 months of follow-up (*P* < 0.001 for all comparisons). However, the HDL particle concentration and HDL subfractions were unaffected by metformin administration.

**Table 3:** Lipoprotein subfractions by treatment group at baseline, 24 hours and 4 months.

	Baseline (Myocardial Infarction)				24 Hours post-MI				4 Months post-MI			
	Placebo (*n* = 186)	Metformin (*n* = 185)	*P*	P*	Placebo (*n* = 172)	Metformin (*n* = 166)	*P*	P**	Placebo (*n* = 160)	Metformin (*n* = 157)	*P*	P***
VLDL particle concentration (nmol/L)	12.2 (3.5–36.5)	15.2 (5.5–42.0)	0.086	0.067	61.4 (45.9–82.7)	64.9 (48.6–84.7)	0.353	0.737	58.3 (44.1–72.9)	62.4 (42.2–81.7)	0.472	0.624
Large VLDL (nmol/L)	0.3 (0.2–0.9)	0.4 (0.2–1.6)	0.117	0.102	6.4 (3.3–10.5)	7.6 (3.9–11.8)	0.175	0.308	4.7 (2.1–8.2)	4.7 (2.1–10.0)	0.096	0.379
Medium VLDL (nmol/L)	0.7 (0.0–4.8)	1.7 (0.0–8.5)	0.242	0.220	27.3 (14.9–39.5)	28.0 (17.5–43.3)	0.642	0.902	22.2 (14.6–33.4)	26.7 (15.0–40.5)	0.112	0.186
Small VLDL (nmol/L)	10.4 (3.0–25.1)	11.3 (4.4–31.3)	0.271	0.247	24.1 (16.3–34.4)	25.4 (15.0–36.8)	0.579	0.594	28.4 (16.7–39.6)	26.0 (16.1–36.4)	0.121	0.047
VLDL Size (nm)	43.8 (37.8–48.1)	44.2 (39.9–50.6)	0.160	0.163	53.4 (47.4–59.4)	54.5 (48.3–60.6)	0.131	0.146	51.7 (46.9–57.2)	53.3 (48.3–59.2)	0.032	0.070
LDL particle concentration (nmol/L)	1509.0 (1292.8–1730.5)	1485.0 (1264.0–1756.0)	0.982	1.000	1262.0 (1055.5–1520.0)	1264.5 (1036.0–1504.5)	0.784	0.729	923.0 (790.5–1114.0)	883.0 (747.0–1048.0)	0.223	0.082
IDL (nmol/L)	102.5 (62.3–180.0)	89.0 (46.0–154.0)	0.080	0.080	81.5 (41.8–146.5)	76.0 (38.0–132.5)	0.295	0.691	40.5 (22.0–69.8)	43.0 (19.0–83.0)	0.713	0.659
Large LDL (nmol/L)	764.5 (546.8–975.0)	665.0 (481.0–898.0)	0.114	0.113	491.5 (330.0–653.3)	432.0 (268.3–597.8)	0.103	0.567	**270.5 (190.0–365.8)**	**170.0 (93.0–278.0)**	**0.000**	**0.000**
Small LDL (nmol/L)	606.5 (423.0–803.8)	659.0 (470.0–923.0)	0.074	0.058	675.0 (496.5–875.0)	708.5 (560.8–959.0)	0.145	0.540	596.5 (470.8–758.8)	631.0 (506.0–795.0)	0.065	0.352
LDL Size (nm)	21.1 (20.7–21.4)	21.0 (20.6–21.3)	0.118	0.112	20.8 (20.4–21.2)	20.7 (20.3–21.0)	0.064	0.297	**20.5 (20.1–20.9)**	**20.3 (20.0–20.6)**	**0.000**	**0.001**
HDL particle concentration (μmol/L)	28.8 (25.6–32.5)	29.4 (26.2–32.5)	0.113	0.111	28.0 (25.0–30.7)	28.0 (24.9–30.7)	0.873	0.201	32.4 (29.1–35.9)	32.6 (28.6–36.1)	0.688	0.403
Large HDL particles (μmol/L)	3.0 (2.1–4.6)	3.1 (2.1–4.9)	0.813	0.781	2.7 (1.7–4.9)	3.1 (1.9–4.7)	0.339	0.305	4.2 (2.6–6.6)	3.8 (2.6–5.8)	0.334	0.675
Medium HDL particles (μmol/L)	14.5 (9.6–20.0)	14.6 (10.1–19.7)	0.903	0.889	9.0 (6.5–11.7)	8.3 (6.0–11.0)	0.293	0.319	9.4 (6.4–12.8)	7.6 (5.5–11.7)	0.012	0.004
Small HDL particles (μmol/L)	9.5 (5.0–14.0)	10.8 (6.3–14.8)	0.266	0.273	15.1 (12.2–18.4)	16.1 (13.1–18.6)	0.559	0.667	17.8 (15.0–21.3)	19.3 (16.7–22.5)	0.022	0.027
HDL Size (nm)	9.0 (8.8–9.3)	9.0 (8.8–9.3)	0.952	0.936	8.7 (8.5–9.2)	8.8 (8.6–9.1)	0.334	0.257	8.9 (8.7–9.3)	8.9 (8.7–9.2)	0.438	0.847

Particle concentrations are presented for subgroups of each major lipid fraction. Size values present the average particle size across all lipoprotein subgroups. Baseline, 24 hours post-MI and 4 months post-MI data are presented as median (interquartile range). *P*-values for placebo vs. Metformin group by unpaired T tests. *P*-value* adjusted for age at randomization, sex, and body mass index. *P*-value** adjusted for baseline value of respective lipoprotein subfractions. *P*-value*** additionally adjusted for statin use at 4 months. Bold is *P*-value ≤ 0.0063.

### Relationship baseline laboratory values with left ventricular ejection fraction and infarct size

Table 4 shows the relationships of baseline laboratory values with LVEF and infarct size at 4 months post-MI. As reported previously [manuscript submitted] NT-proBNP at baseline was negatively associated with LVEF 4 months post-MI (*P* = 0.008). Glucose level at baseline was positively associated with infarct size (*P* = 0.001). After adjustment for age, sex, baseline NT-proBNP level, treatment allocation and myocardial blush grade this relationship remained significant (*P* = 0.003).

**Table 4:** Relationship LVEF and infarct size at 4 months with baseline laboratory values.

	LVEF β (95% CI)	*P*	P*	Infarct Size β (95% CI)	*P*	P*
Glucose	-1.024 (-2.051, 0.002)	0.050	0.073	**1.577 (0.640, 2.514)**	**0.001**	**0.003**
HbA1c	0.551 (-0.513, 1.615)	0.309	0.172	-0.230 (-1.219, 0.760)	0.648	0.417
Total Cholesterol	-0.308 (-1.329, 0.712)	0.552	0.367	0.279 (-0.685, 1.243)	0.570	0.337
LDL Cholesterol	-0.288 (-1.306, 0.730)	0.578	0.278	0.198 (-0.762, 1.158)	0.685	0.379
non-HDL Cholesterol	-0.147 (-1.163, 0.869)	0.776	0.468	0.001 (-0.951, 0.953)	0.998	0.577
HDL Cholesterol	-0.385 (-1.424, 0.653)	0.466	0.840	0.806 (-0.170, 1.782)	0.105	0.272
Triglycerides	0.520 (-0.626, 1.667)	0.372	0.544	-0.509 (-1.589, 0.571)	0.354	0.730
ApoB	-0.381 (-1.417, 0.656)	0.470	0.475	-0.069 (-1.072, 0.935)	0.893	0.846
ApoA-I	-0.448 (-1.416, 0.521)	0.364	0.494	0.255 (-0.670, 1.180)	0.588	0.701
NT-proBNP	**-1.383 (-2.405, -0.362)**	**0.008**		0.745 (-0.217, 1.707)	0.128	

Linear regression model of LVEF or infarct size with baseline laboratory values. Unadjusted coefficients are shown. Abbreviations: Apo: apolipoprotein; HbA1c: glycosylated hemoglobin; HDL: high density lipoproteins; LDL: low density lipoproteins; LVEF: left ventricular ejection fraction; non-HDL: non-high density lipoproteins; NT-proBNP: N-terminal pro brain natriuretic peptide. *P*-value*: adjusted for age at randomization, sex, baseline NT-proBNP concentration, treatment allocation, myocardial blush grade and statin use at 4 months. Bold is *P*-value ≤ 0.01.

### Relationship of lipoprotein subfraction levels at baseline and 24 Hours post-MI with left ventricular ejection fraction and infarct size

Table 5 shows the relationships of lipoprotein subfraction levels at baseline and 24 hours with LVEF and infarct size at 4 months after MI. None of the lipoprotein subfractions at baseline were associated with LVEF or infarct size. The concentration of small HDL particles 24 hours post-MI was positively associated with LVEF (*P* = 0.003) and negatively with infarct size (*P* = 0.006). Furthermore, the total VLDL particle concentration (*P* = 0.003) and the medium VLDL particle concentration (*P* = 0.001) 24 hours post-MI were both negatively associated with infarct size. After adjustment for age, sex, baseline NT-proBNP level, treatment allocation, myocardial blush grade and statin use, the relationship between small HDL particles and LVEF (*P* = 0.005), and between the medium VLDL particle concentration with infarct size (*P* = 0.001) remained significant. The association of the total VLDL particle concentration and of small HDL particles with infarct size was not significant after adjustment for these covariates.

**Table 5:** Relationship of LVEF and infarct size at 4 months with lipoprotein subfraction levels at baseline and 24 hours.

	Baseline (Myocardial Infarction)						24 hours post Myocardial Infarction					
	LVEF β (95% CI)	*P*	P*	Infarct Size β (95% CI)	*P*	P*	LVEF β (95% CI)	*P*	P*	Infarct Size β (95% CI)	*P*	P*
VLDL particle concentration	0.843 (-0.213, 1.900)	0.117	0.152	-0.573 (-1.553, 0.407)	0.251	0.531	1.719 (0.333, 3.105)	0.015	0.015	**-1.875 (-3.107, -0.643)**	**0.003**	0.008
Large VLDL	0.257 (-0.942, 1.456)	0.673	0.601	-0.339 (-1.440, 0.762)	0.545	0.812	1.034 (-0.397, 2.466)	0.176	0.171	-1.278 (-2.567, 0.010)	0.052	0.086
Medium VLDL	0.852 (-0.384, 2.088)	0.176	0.179	-1.151 (-2.291, -0.012)	0.048	0.100	1.731 (0.374, 3.089)	0.012	0.007	**-2.186 (-3.407, -0.965)**	**0.001**	**0.001**
Small VLDL	0.667 (-0.229, 1.563)	0.144	0.190	-0.278 (-1.120, 0.565)	0.517	0.862	0.284 (-1.022, 1.590)	0.653	0.864	-0.089 (-1.276, 1.099)	0.883	0.820
VLDL Size	0.086 (-1.098, 1.270)	0.886	0.866	-0.294 (-1.379, 0.791)	0.594	0.701	0.550 (-0.741, 1.841)	0.399	0.394	-0.509 (-1.663, 0.644)	0.385	0.431
LDL particle concentration	-0.078 (-1.257, 1.101)	0.896	0.586	0.076 (-1.027, 1.178)	0.893	0.549	0.431 (-0.816, 1.677)	0.495	0.574	-0.314 (-1.438, 0.810)	0.583	0.799
IDL	-0.075 (-1.151, 1.001)	0.891	0.796	-0.742 (-1.741, 0.258)	0.145	0.153	0.595 (-0.456, 1.646)	0.265	0.487	-0.234 (-1.195, 0.727)	0.632	0.903
Large LDL	-0.996 (-2.063, 0.070)	0.067	0.028	1.069 (0.073, 2.065)	0.035	0.029	-0.438 (-1.720, 0.844)	0.500	0.212	1.076 (-0.097, 2.248)	0.072	0.041
Small LDL	0.762 (-0.138, 1.663)	0.097	0.101	-0.614 (-1.451, 0.223)	0.150	0.263	0.513 (-0.538, 1.563)	0.337	0.153	-0.885 (-1.825, 0.056)	0.065	0.064
LDL Size	-1.065 (-2.174, 0.045)	0.060	0.024	1.125 (0.094, 2.155)	0.033	0.034	-0.432 (-1.526, 0.662)	0.520	0.147	0.974 (-0.002, 1.949)	0.050	0.029
HDL particle concentration	0.185 (-0.896, 1.265)	0.737	0.462	-0.525 (-1.533, 0.483)	0.306	0.211	1.317 (0.110, 2.524)	0.031	0.051	-0.887 (-1.986, 0.211)	0.113	0.149
Large HDL particles	0.286 (-0.784, 1.357)	0.599	0.315	0.462 (-0.536, 1.460)	0.363	0.752	-0.264 (-1.301, 0.773)	0.632	0.661	0.335 (-0.599, 1.269)	0.480	0.740
Medium HDL particles	0.207 (-0.867, 1.280)	0.705	0.782	-0.542 (-1.542, 0.458)	0.287	0.377	-0.651 (-2.030, 0.727)	0.352	0.352	0.594 (-0.641, 1.829)	0.344	0.244
Small HDL particles	-0.264 (-1.382, 0.854)	0.642	0.888	0.127 (-0.922, 1.177)	0.811	0.985	**2.315 (0.815, 3.814)**	**0.003**	**0.005**	**-1.913 (-3.273, -0.554)**	**0.006**	0.009
HDL Size	0.414 (-0.853, 1.681)	0.520	0.275	0.503 (-0.686, 1.693)	0.405	0.780	-0.439 (-1.432, 0.555)	0.505	0.644	0.367 (-0.530, 1.264)	0.421	0.670

Linear regression model of left ventricular ejection fraction (LVEF) or infarct size with lipoprotein subfractions as Baseline (MI) and 24 hours post-MI. Unadjusted β coefficients are shown. *P*-value is from univariate linear regression model. *P*-value* is from adjusted model for age at randomization, sex, baseline NT-proBNP concentration, treatment allocation, myocardial blush grade and statin use at 4 months. Bold is *P*-value ≤ 0.0063.

## Discussion

We show here that 4 months metformin administration initiated directly after the acute phase of MI is associated with slight reductions reduced LDL cholesterol without affecting apoB levels in a large group of patients without previously established diabetes. Metformin decreased large LDL particles by approximately 35% without a significant effect on small LDL particles. Consequently, LDL size was also decreased. Metformin did not significantly affect plasma triglycerides, VLDL characteristics, HDL cholesterol, apoA-I and HDL subfractions. In addition, we observed that small-sized HDL particles and medium-sized VLDL obtained after 24 hours were associated with higher LVEF and a smaller infarct size.

The present findings on plasma (apo)lipoproteins and lipoprotein subfraction characteristics should be interpreted in the context of lipoprotein changes that occur in the setting of an acute coronary syndrome [10,31]. Thus, LDL cholesterol spontaneously decreases shortly after MI and rises again after several weeks [10,31]. In the current study, the LDL particle concentration was lower at 24 hours post-MI in both treatment groups, which could to at least in part be attributed by the initiation of statin therapy in the vast majority of study participants. In addition, initiation of statin treatment largely explained lower levels of total cholesterol, LDL cholesterol, non-HDL cholesterol, apoB and LDL subfractions after 4 months. These lipoprotein changes were present irrespective of metformin treatment. Plasma triglycerides may acutely decrease after an MI, followed by an increase above baseline after several days [10]. All participants received heparin before percutaneous coronary intervention mostly before arrival at the hospital as part of routine medical care [32]. It is well known that heparin stimulates lipoprotein lipase, thereby increasing lipolysis [33]. Even a low dose of heparin lowers plasma triglycerides acutely [34]. This explains our observation that plasma triglycerides were about 50% lower at presentation compared to 4 months follow-up. The VLDL particle concentration was even 5-fold lower at baseline vs, the levels obtained after 24 hours and after 4 months. On the other hand, HDL cholesterol concentration has been reported to remain fairly constant during the acute phase of MI [10,31]. Accordingly, we did not observe much change in the HDL particle concentration and in HDL subfraction levels 24 hours after manifestation of MI.

The effects of metformin when initiated during the acute phase of MI to reduce LDL cholesterol and large LDL particles has not been described in previously. In comparison, one year treatment with metformin dosed 850 mg administered twice daily decreases the LDL particle concentration, small-sized LDL particles and slightly increases LDL size along with improvement in insulin sensitivity in subjects with impaired glucose tolerance [22]. While metformin increases small HDL particles in the non-acute setting [22], we did not found a change in small HDL particles in the present study. It has also been shown that 18 months treatment with metformin at a dose of 850 mg twice daily does not significantly affect plasma total cholesterol, LDL cholesterol, HDL cholesterol and triglycerides despite improvement in insulin sensitivity in statin-using non-diabetic subjects with stable coronary heart disease [23]. These variable results emphasize the relevance of participant selection, the circumstances of initiation of metformin treatment and possibly also of its dose and exposure time.

In the general population, an inverse relationship of HDL cholesterol with incident coronary heart disease has been consistently reported [35,36]. Moreover, the relevance of HDL subfractions for cardiovascular risk prediction has received considerable attention [6,9,12,17,28]. As yet, the importance of larger-sized compared to smaller-sized HDL particles for coronary risk has not been unequivocally established, neither in the setting of population-based cohort studies [9,12,17,28], nor in specific high risk populations [3,4,37]. Of further interest, low HDL cholesterol as determined during an acute coronary syndrome may predict recurrent cardiovascular events [38]. More recently, it was reported that HDL-associated cholesterol esterification is impaired in the acute setting of a coronary syndrome despite a lack of decrease in HDL cholesterol [39], whereas HDL anti-inflammatory function rather than HDL cholesterol may predict recurrent events [40]. In non-acute patients, impaired ability of HDL to remove cholesterol from macrophage model cells associates more closely with incident coronary heart disease than lower HDL cholesterol [41]. These findings emphasize the importance of HDL functional properties for atheroprotection [10,42]. In the context of the GIPS-III trial it is also relevant that a low HDL cholesterol concentration may represent a determinant of heart failure [43]. Experimental induction of apoA-I, HDL’s most abundant apolipoprotein, improves cardiac remodeling after MI in mice [44], although HDL mimetic therapy was unsuccessful in improving cardiac outcome in humans [45]. In coronary artery disease patients, it has been cross-sectionally determined that HDL cholesterol and smaller-sized HDL may confer higher LVEF [3,46]. In the current report, we considered the metformin- and placebo-receiving participants together since metformin did not affect LVEF [27]. After adjustment for treatment allocation and other relevant covariates we demonstrated that increased concentrations of smaller-sized HDL particles prospectively predict higher LVEF. These novel results agreement with the concept that specific HDL subfractions could be pathophysiologically implicated in better cardiac performance. The mechanisms responsible for this association remain to be more precisely delineated. Among other possibilities, it could reflect the ability of certain HDL subfractions to exert anti-oxidative properties or to stimulate endothelial function [10,26,42]. In addition, higher concentrations of medium VLDL were associated with smaller infarct size. As yet the clinical implication of this association is unknown.

Several other methodological aspects of our study need to be considered. First, this randomized study was carried out in a considerable number of participants, making lack of power to demonstrate effects of metformin on VLDL and HDL subfraction characteristics as determined by NMR spectroscopy unlikely. Moreover, neither plasma triglycerides nor HDL cholesterol and apoA-I levels after 4 months of follow-up changed in response to metformin administration. Second, inherent to the design of this study to randomize subjects shortly after arrival at the hospital, plasma lipid measurements were not carried out in the fasting state. For logistic reasons, non-fasting samples were also obtained during follow-up. However, given the placebo-controlled design of GIPS-III, it is unlikely that this approach materially affected our results. Third, only 2% of participants experienced recurrent major adverse cardiac events, and none of participants died during 4 months follow-up [27]. For this reason, associations of lipoprotein subfractions with hard clinical end-points could not be assessed. Instead, LVEF at 4 months follow-up was chosen as the pre-specified primary endpoint of the GIPS-III trial, reasoning that left ventricular dysfunction is a prevalent complication of STEMI which prospectively predicts poor cardiac outcome [26,27]. Fourth, only subjects without known diabetes participated in the GIPS-III trial [18,19]. The positive correlation between glucose at admission and MI size after 4 months, therefore, suggests that MI size may relate to stress hyperglycemia encountered during the acute phase of MI [47].

In conclusion, the present study suggests that metformin treatment initiated directly after the acute phase of MI elicits a small decrease in LDL cholesterol together with a decrease in LDL size. Furthermore, higher medium VLDL and higher small HDL particle concentrations may confer beneficial associations with increased LVEF and decreased infarct size, respectively.

## Supporting Information

S1 Fig Correlation matrix using Pearson correlation coefficients from the metabolite concentrations of the lipoprotein subfractions 4 months post-MI (*n* = 317). (TIFF)

S2 Fig Panel showing changes in median of metabolite levels over the three time points Myocardial Infarction (MI), 24 hours post-MI (24h) and 4 months post-MI (4M) on the X- axis. Red lines represent placebo and blue lines metformin treatment. The Y-axis represents the metabolite concentration with units given in either nmol/L, μmol/L, or nm.
(TIFF)

S1 File Overview of Principal Components (PC) analysis of the lipoprotein subfractions showing the importance of the components. (XLSX)
