# Supplementary figures and images for: Effect of Metformin Treatment on Lipoprotein Subfractions in Non-Diabetic Patients with Acute Myocardial Infarction: A Glycometabolic Intervention as Adjunct to Primary Coronary Intervention in ST Elevation Myocardial Infarction (GIPS-III) Trial
Source: PLoS One. 2016 Jan 25;11(1):e0145719. doi: 10.1371/journal.pone.0145719 (PMC4726568; doi:10.1371/journal.pone.0145719)

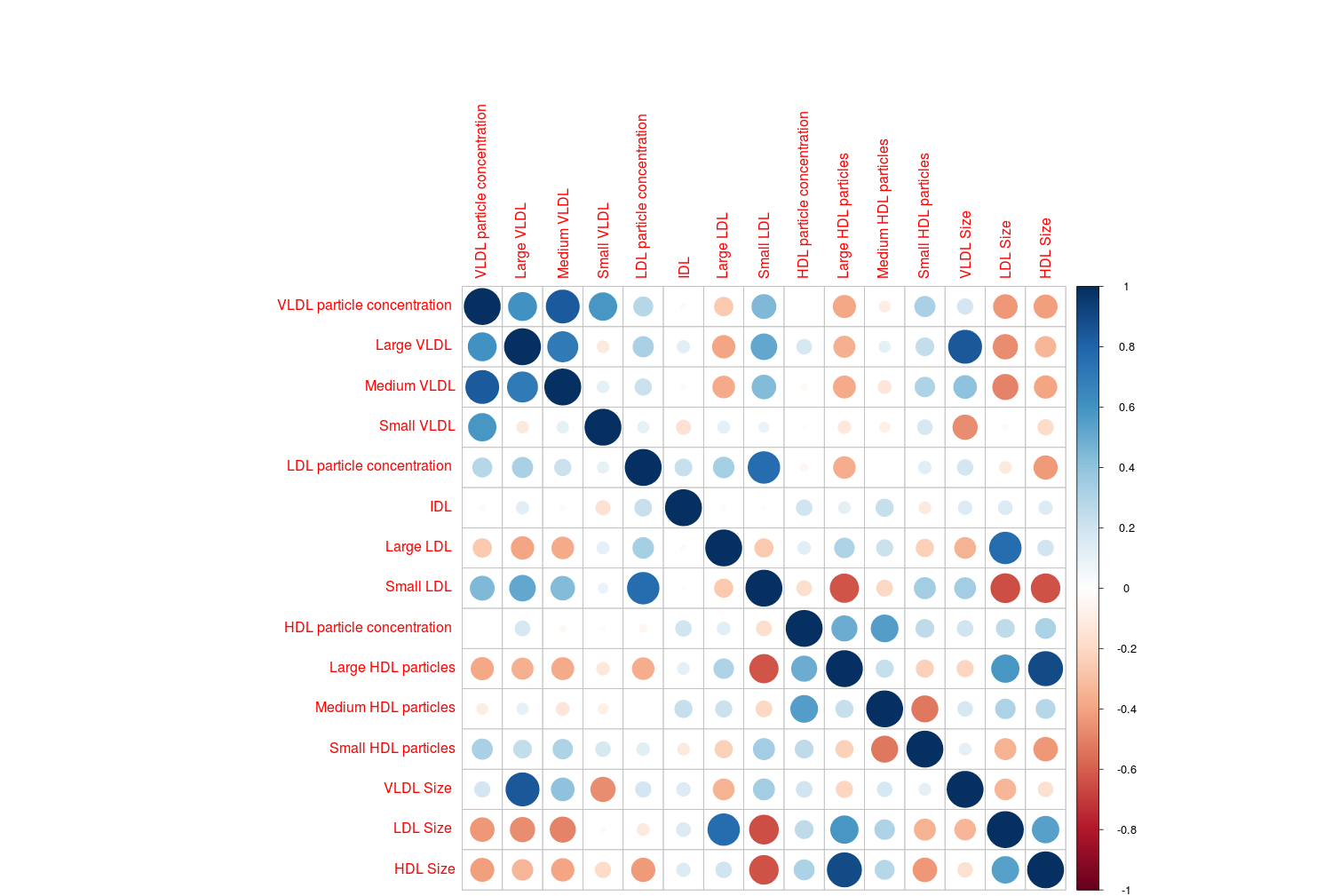

Supplement: S1 Fig — (TIFF) [file pone.0145719.s001.tiff]

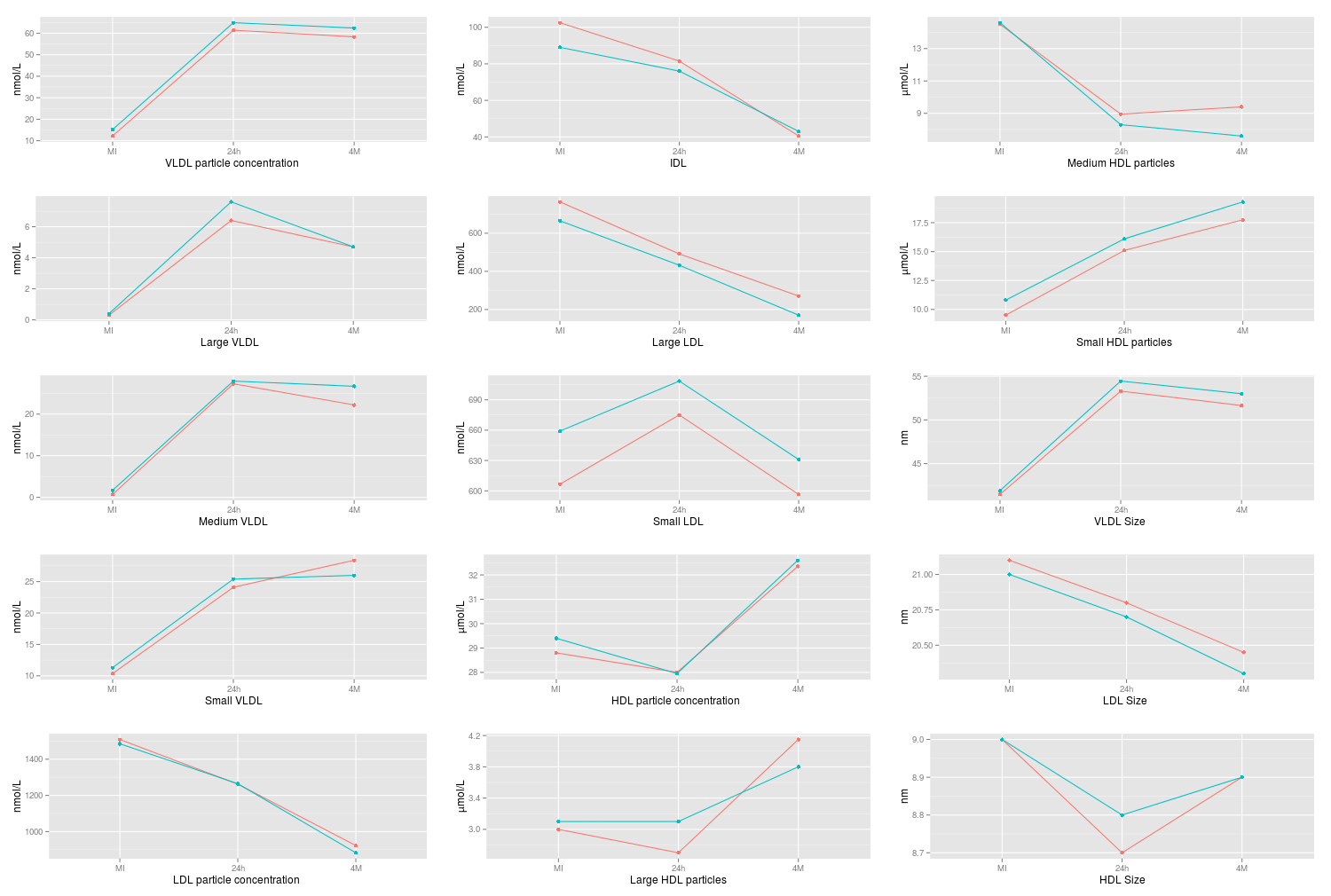

Supplement: S2 Fig — Red lines represent placebo and blue lines metformin treatment. The Y-axis represents the metabolite concentration with units given in either nmol/L, μmol/L, or nm. (TIFF) [file pone.0145719.s002.tiff]
